# Reexamining cholinesterase inhibitors for the prevention and treatment of delirium in high-risk populations

**DOI:** 10.1186/s13054-023-04413-w

**Published:** 2023-03-31

**Authors:** Ori J. Lieberman, Vanja C. Douglas, Sara C. LaHue

**Affiliations:** 1 Department of Neurology, University of California, San Francisco, CA USA; 2 Buck Institute for Research on Aging, Novato, CA USA

We read with interest the article Hughes et al. recently published in *Critical Care* describing the correlation between circulating levels of acetylcholinesterase (AChE) and butyrylcholinesterase (BChE) activity in critically ill adults and their risk of delirium or coma [1]. The implementation of a point-of-care testing paradigm for serum AChE and BChE activity raises the possibility of identifying a population at high risk of developing delirium for future clinical trials.

Delirium is a state of acute brain failure seen commonly in critically ill individuals, variably associated with inattention, positive symptoms such as hallucinations or delusions, and altered level of consciousness, for which no treatment exists [2]. Although the pathophysiology of delirium remains obscure and complex, dysfunction of central neuromodulatory transmission by acetylcholine, norepinephrine and dopamine contributes to the development of the key behavioral manifestations of delirium [2]. High levels of circulating pro-inflammatory cytokines are thought to impair cholinergic neurotransmission during critical illness, possibly acting as a final common pathway in the development of this syndrome [2]. Enhancing cholinergic neurotransmission during episodes of delirium has been proposed as an approach to prevent and treat delirium during critical illness [3].

Cholinergic deficits are also thought to underlie cognitive symptoms in adults with several types of dementias, including Alzheimer’s disease, dementia with Lewy bodies, and vascular dementia [4], and these individuals are at high risk for developing delirium during hospitalization [2]. There is widespread clinical use of acetylcholinesterase inhibitors (AChEi), which block the enzyme that degrades synaptic acetylcholine, in people with dementia. AChEi’s, like donepezil and rivastigmine, provide symptomatic benefit in neurocognitive disorders by reducing the slope of cognitive decline and improving functional independence [5]. Their use is generally well-tolerated, with major side effects including diarrhea and bradycardia. Importantly, large registry studies demonstrated reduced cardiovascular and cerebrovascular events, as well as incident mortality, in people with dementia prescribed AChEis in the outpatient setting [6, 7].

Several randomized controlled trials have evaluated the ability of AChEi to prevent or treat delirium [8–13]; however, none has tested these medicines in a critically ill population enriched for preexisting central cholinergic deficits, such as those with dementia. Early studies that focused on prevention of delirium prior to an elective operation showed minimal benefit, although these were of varying methodological rigor [8, 9, 13, 14]. Unfortunately, a large trial comparing behavioral management with haloperidol and placebo versus haloperidol and rivastigmine in a general population of critically ill adults was stopped early due to a signal suggesting increased mortality in the rivastigmine group; however, differences in baseline characteristics between the groups call into question whether this would have been borne out in a fully powered trial [11]. One single prospective study tested whether rivastigmine would prevent post-operative delirium in a population with preexisting cognitive impairment [10]. This study, although small, suggested an impressive risk reduction in incident delirium and delirium severity. Whether these benefits would translate to a critically ill population with preexisting cognitive impairment remains unknown.

In support of the hypothesis that patients with preexisting cognitive impairment would benefit from AChEi during critical illness, we recently reported a retrospective analysis of the MIMIC database of nearly 60,000 ICU admissions at Beth Israel Deaconess Medical Center in Boston [15]. When older adults with preexisting dementia were stratified based on whether they were prescribed donepezil before admission, those receiving donepezil had lower rates of incident delirium, in-hospital mortality, 90-day post-discharge mortality, lengths of stay and duration of mechanical ventilation, despite similar illness severity and comorbidities at admission. Causal inference methods demonstrated that the reduction in incident delirium mediated donepezil’s effect on length of stay and duration of mechanical ventilation.

We propose a reexamination of the role for AChEi’s in delirium prevention and treatment during critical illness (Fig. 1). Given the broad range of predisposing factors and precipitants for delirium among critically ill patients [14], careful study population selection, including those with preexisting cognitive impairment or biomarkers of reduced cholinergic signaling, may maximize the potential for detecting a clinically relevant benefit. Designing such a clinical trial would necessitate identifying a population at high risk for developing delirium. At ICU admission, the use of rapid point-of-care testing for the measurement of serum AChE and BChE activity, such as that reported by Hughes et al. [1], or selection of those with co-existing cognitive impairment [15], could be used to further enrich a population with a cholinergic deficit. Randomization to AChEi initiation or placebo could then occur and in-hospital and post-discharge outcomes would be collected. Altogether, such studies would definitively test whether AChEi’s, medicines already in widespread clinical use and with substantial mechanistic support, would provide a benefit in the prevention or treatment of delirium during critical illness.

**Fig. 1:**
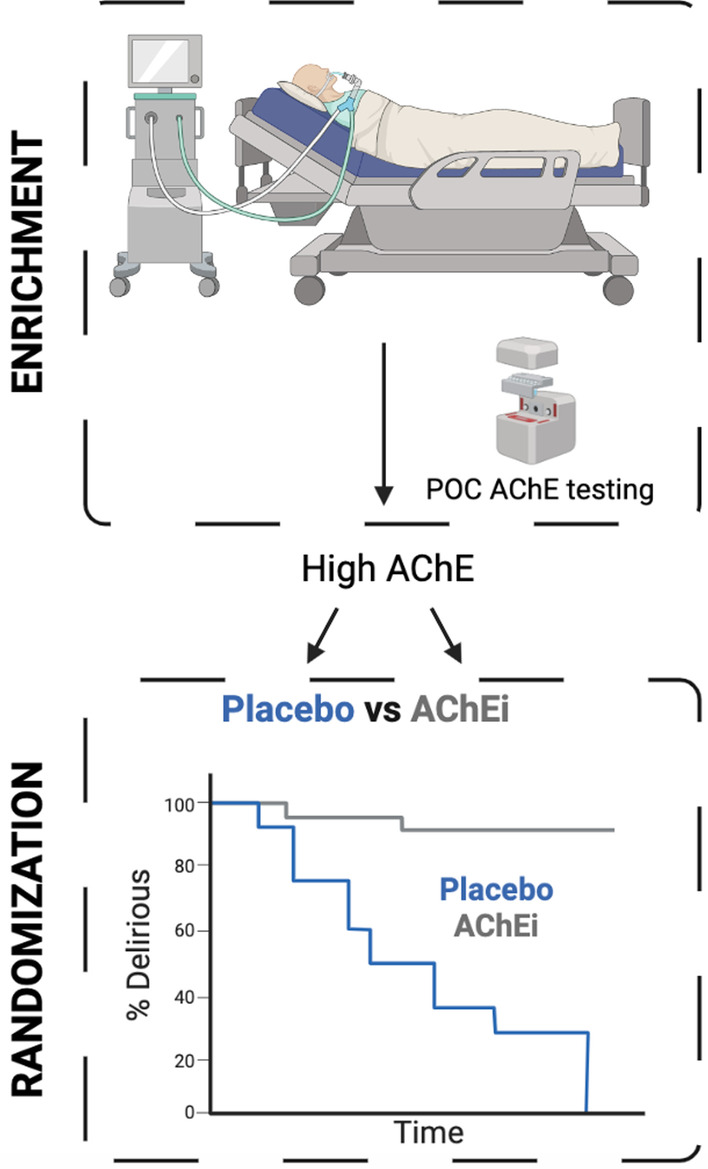
Proposed study design incorporating point-of-care testing in patients with baseline dementia to test whether AChEi may reduce the risk of delirium during critical illness. Designed on Biorender

We look forward to the next generation of studies that employ novel molecular diagnostics in high-risk populations to identify new treatments that can prevent or treat delirium during critical illness and improve outcomes among these vulnerable individuals.

## Data Availability

Not applicable.
